# Unveiling the Potential of Bioactive Glass in Volumetric Muscle Loss Regeneration

**DOI:** 10.3390/ma18112529

**Published:** 2025-05-27

**Authors:** Andreea-Alina Zăvoi, Alexandra Dreancă, Klara Magyari, Lucian Baia, Ciprian Ober, Liviu Oana

**Affiliations:** 1 Faculty of Veterinary Medicine, University of Agricultural Science and Veterinary Medicine, 400372 Cluj-Napoca, Romania; andreea-alina.zavoi@usamvcluj.ro (A.-A.Z.); ciprian.ober@usamvcluj.ro (C.O.); liviu.oana@usamvcluj.ro (L.O.); 2 Nanostructured Materials and Bio-Nano-Interfaces Center, Interdisciplinary Research Institute on Bio-Nano-Sciences, Babes-Bolyai University, 400084 Cluj-Napoca, Romania; lucian.baia@ubbcluj.ro; 3 INSPIRE Research Platform, Babes Bolyai University, 400084 Cluj-Napoca, Romania; 4 Faculty of Physics, Babes Bolyai University, 400084 Cluj-Napoca, Romania

**Keywords:** volumetric muscle loss, tissue engineering, skeletal muscle regeneration, bioactive glasses

## Abstract

Injuries characterized by significant loss of skeletal muscle tissue volume, known as volumetric muscle loss (VML), lead to substantial impairment in functional capabilities. Natural repair processes and existing medical interventions fall short of fully restoring function post-VML. Despite progress in the VML field, there is an unsatisfactory success rate, donor site morbidity, and inefficient reconstruction of lost muscle tissue. This leads to persistent strength and functional deficits, impacting the quality of life for VML patients. In recent years, studies have explored the potential of bioactive glasses (BGs) as crucial materials in regenerating tissues beyond the skeletal system. BG, used mainly in bone engineering, can aid muscle repair by releasing ions like calcium and phosphate to stimulate cellular response. However, current BG composites struggle to match the mechanical properties of soft tissues, limiting seamless healing. This review summarizes recent advances in various BG structures studied for skeletal muscle tissue regeneration.

## 1. Overview

This review provides a structured analysis of the potential of bioactive glasses (BGs) in skeletal muscle regeneration, particularly in the context of volumetric muscle loss (VML). The key topics covered include the following:

- An overview of skeletal muscle structure and function;
- The natural muscle regeneration process and its limitations in VML;
- The potential of bioactive glasses (BG-) for skeletal muscle tissue engineering;
- BG properties, mechanisms of action, and bioactivity;
- Recent advances and studies on BG applications in muscle regeneration;
- Key findings and ongoing challenges in the field;
- Future perspectives on BG-based strategies for treating VML injuries.

## 2. Introduction

Skeletal muscles in humans represent 40% of the body weight and have multiple functions, i.e., they convert chemical energy into mechanical energy and actively contribute to physical movements [1]. Volumetric muscle loss (VML) is a specific type of muscle injury defined by the significant loss of 20% or more of the total muscle tissue in a three-dimensional volume. Unlike minor muscle injuries that primarily affect the muscle fibers themselves, VML involves the substantial loss of muscle fibrils and also the surrounding connective tissue, blood vessels, nerves, and other components that make up a functional muscle unit [2].

Severe limb injuries in orthopedics often result in a volumetric loss of skeletal muscle, causing chronic muscle weakness, impaired limb function, and disability [2,3]. Such injuries may be primary or secondary. The primary injury can occur initially from trauma, for example, after a car accident or through trauma sustained by soldiers on a mission. Secondary lesions occur after the tissue adjacent to the primary lesions deteriorates, so the damaged/contaminated muscle must be debrided or evacuated [4]. Recently, there has been more focus on studying VML injuries in musculoskeletal trauma, driven by the increased frequency of such injuries among victims in recent wars [2,3,5,6]. Considering the nature of the injury, which involves a substantial loss of muscle tissue surpassing the regenerative capacity of the remaining musculature, current research endeavors concentrate on developing therapies to regenerate new muscle tissue, aiming to restore muscle strength [7,8,9]. VML injuries are often seen in the lower limb, with quadriceps VML being particularly noteworthy [6].

**Figure 1:**
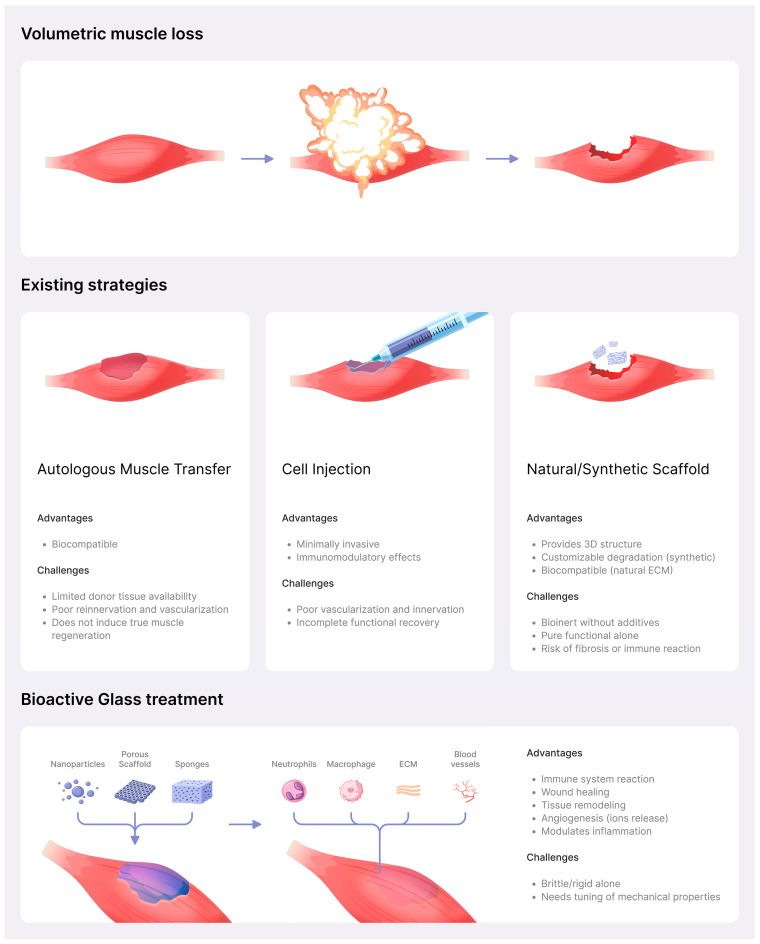
The process of VML and the regeneration strategies.

The aim of this review article is to provide an overview of the role of biomaterials containing bioactive glasses (BGs) in addressing VML, highlighting their potential in facilitating muscle regeneration and treating musculoskeletal defects. The first part will describe the skeletal muscle regeneration process and its limitations, followed by an overview of BG as a potential material for skeletal muscle tissue regeneration, including some results from the literature (Figure 1).

## 3. Skeletal Muscle Structure and Function

The structural layout of skeletal muscle is distinguished by a clearly defined arrangement of muscle fibers, referred to as myofibers or muscle cells, accompanied by surrounding connective tissue. At the macroscopic level, the size of a muscle is primarily determined by the quantity and size of individual muscle fibers, although pathological infiltration by fat and connective tissue can affect this relationship [10,11]. Muscle fibers are multinucleated and post-mitotic. Typically, each nucleus within a muscle fiber governs the synthesis of specific proteins in its designated region, known as nuclear domains, which are highly regulated but not constant in size [12]. Protein expression in adjacent domains of a single fiber is usually coordinated to produce similar types of proteins, such as myosin, along the fiber’s length. However, occasional non-uniformity in contractile properties across adjacent segments of a single fiber has been observed [13]. Satellite cells serve as the adult stem cells of skeletal muscle, located between the sarcolemma and the basal lamina. They play crucial roles in muscle growth, repair, and regeneration. When activated by myogenic factors, satellite cells undergo proliferation and differentiation to form new muscle fibers [12,14,15].

Each myofiber is enveloped by a basal lamina and, beyond this, by the endomysium, a thin layer of connective tissue housing capillaries. These myofibers are organized into fascicles, surrounded by the perimysium, which is a slightly denser layer of connective tissue. The muscle is enclosed within the epimysium, a protective fascia that blends with the muscle tendon. This framework of connective tissue is not inert; rather, it plays an integral role in muscle contractile function by storing and transmitting the force generated by myofiber contraction. Near the sarcolemma, there exists a network of multiple proteins (Z disk) physically linked to the internal myofilament structure, especially to the actin protein found in the thin filament. Partial or complete absence or dysfunction of any of these proteins can damage the sarcolemma, causing muscle weakness and atrophy. For instance, within this complex resides the protein dystrophin, which is either partially or entirely lacking in certain neuromuscular disorders like Duchenne and Becker muscular dystrophies [16].

**Figure 2:**
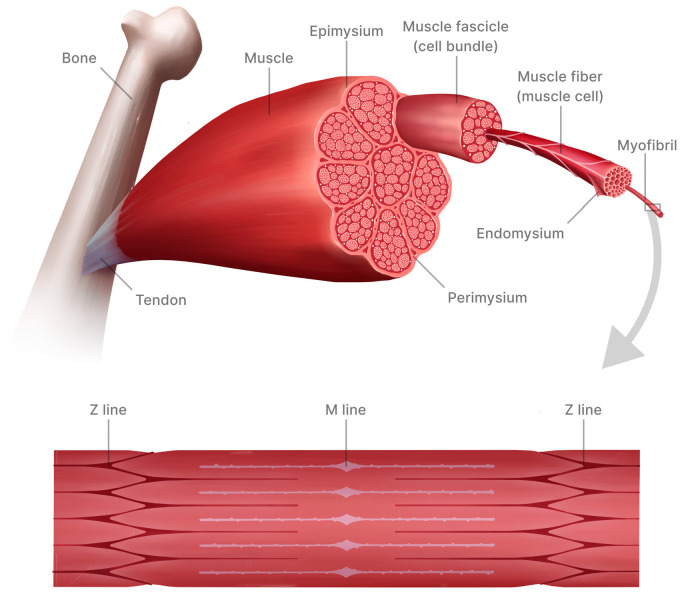
Schematic representation of skeletal muscle structure. The muscle is anchored to the bone through a tendon and enclosed within the epimysium. Internally, bundles of muscle fascicles are surrounded by perimysium, while each individual muscle fiber (cell) is wrapped in endomysium. Muscle fibers contain myofibrils composed of serially arranged sarcomeres, delineated by Z lines and M lines. This hierarchical organization underlies the ability of skeletal muscle to generate contraction and produce force. Adapted with permission from [20]. Copyright (2018) with permission from McGraw-Hill Education.

Skeletal muscle is made up of functional units called motor units, which consist of a group of muscle fibers and the motor neuron that innervates them. Within each motor unit, the muscle fibers display comparable contractile and biochemical characteristics largely governed by the motor neuron. Through biochemical methods and histochemical staining, muscle fibers are typically categorized as red (type I, slow-twitch, oxidative, dark-staining) or white (type II, fast-twitch, glycolytic, light-staining). In rodents, individual skeletal muscles often display a high level of uniformity [17,18]. However, this uniformity is not consistently observed in humans, where differences in fiber type composition between individuals may account for significant variations in metabolic potential and exercise capacity seen among athletes (Figure 2) [19].

The preservation of muscle mass relies on a delicate interplay between protein synthesis and degeneration, a balance influenced by various factors such as nutrition, hormonal levels, activity levels, and health status. The different protein compartments within muscles—structural, contractile, and regulatory—have captured considerable scientific interest due to their critical roles in mobility, exercise capacity, functionality, and overall well-being.

As one of the most prominent and important parts of the body, skeletal muscle serves numerous functions within the body, maintaining posture and enabling movement, including locomotion. Additionally, muscles play a significant role in maintaining whole-body homeostasis; and from a purely aesthetic view, muscles contribute to the pleasing contours of the body.

From a metabolic standpoint, skeletal muscle contributes to basal energy metabolism, serves as a storage site for essential substrates like amino acids and carbohydrates, generates heat for core temperature regulation, and consumes a substantial portion of oxygen and fuel during physical activity [21]. Of particular importance is its role as a storage for amino acids essential in synthesizing tissue-specific proteins found in organs such as the skin, brain, and heart. Additionally, the release of amino acids from muscle aids in maintaining blood glucose levels during periods of starvation. Importantly, diminished muscle mass compromises the body’s ability to cope with stress and chronic illness, thereby underscoring its significance in disease prevention and health maintenance [22].

## 4. Skeletal Muscle Regeneration Process and Its Limitations

The musculoskeletal system is made up of bones, muscles, and connective tissues. Its primary role is to provide structural support, facilitate movement, protect internal organs, and allow locomotion to mammals [23]. Mechanical stress is well recognized as the predominant aetiology of muscle injury [24]. Despite recurrent trauma, skeletal muscle has a remarkable regenerative capability, with full-function recovery often restored within 21 days and the ability to re-innervate [25]. The process of skeletal muscle repair involves several distinct phases that collectively aim to restore damaged muscle tissue [26,27]. These phases are characterized by a sequence of events that work in a coordinated manner to facilitate healing and functional recovery.

- Inflammatory phase: Damaged muscle fibers release cellular contents into the surrounding tissue, triggering an inflammatory response where immune cells are recruited to the injury site to remove cellular debris and begin the regeneration process. In response to injury, satellite cells (a population of muscle-specific stem cells) become activated.
- Proliferation phase: Cells proliferate and migrate to the site of injury to form a temporary extracellular cell matrix, guided by chemical signals [27]. This is followed by the regeneration phase, in which satellite cells differentiate into myoblasts that fuse into new myotubes, integrating with existing muscle fibers. Simultaneously, blood vessels regenerate (neoangiogenesis) to supply nutrients.
- Remodeling phase: The final phase, remodeling/maturation, involves the realignment of muscle fibers and the deposition of connective tissue to enhance structural integrity. The formation of functional neuromuscular junctions is essential for muscle contraction [3]. It is important to note that while this sequence of events generally characterizes the muscle repair process, the speed and efficacy of each phase can vary depending on factors such as the extent of injury, individual health, and age. However, this intrinsic regenerative response is often insufficient for substantial muscle loss, necessitating advanced approaches [28].

Currently, there is a lack of established clinical guidelines for addressing VML wounds, leading to limited treatment options and demanding novel strategies to restore function and strength. To overcome these barriers, researchers have been developing diverse bioengineering methods for new and improved therapies for musculoskeletal disorders.

Clinical treatments used for VML encompass a spectrum of strategies, each with its distinct strengths and limitations. Autologous tissue reconstruction and allogeneic grafts are commonly employed to replace lost muscle mass. While these strategies can provide structural support, they might not completely restore the original function. Moreover, these procedures often entail donor site morbidity, limited graft availability, and immune-related challenges. Approximately 10% of graft failures are attributed to issues like venous clotting, blockage of arteries, infections, and mechanical strain around the surgical connection [29,30,31].

With a growing need for treatments that can fully restore skeletal muscle after severe trauma, researchers are focusing on tissue engineering treatments. To effectively guide muscle regeneration, biomaterials designed for skeletal muscle tissue engineering must possess several desirable properties, taking into account the limitations of current therapies. Some of the crucial characteristics include porosity, aligned structure, and the incorporation of biochemical signals.

## 5. Bioactive Glasses as Potential Materials for Skeletal Muscle Tissue Regeneration

The tissue engineering and regenerative medicine approach for VML involves the utilization of various biomaterials, cells, growth factors, and engineering techniques to promote healing and functional recovery of the compromised tissue [32]. These approaches aim to create a conducive environment that supports the growth, differentiation, and integration of muscle cells, as well as the development of blood vessels and nerve innervation [33]. The prospect of using biodegradable scaffolds emerges as a promising pathway in treating the loss of muscle tissue, as they possess the potential to replace the absent structural framework. Various types of scaffolds have been explored for VML treatment, resulting in varying degrees of success [34,35,36,37,38,39]. While certain materials like decellularized tissue have exhibited achievements in promoting viable tissue regeneration, researchers have also documented the undesirable formation of scar tissue and inadequate enhancements in post-implantation recovery [40].

BG are one of the promising materials with the capability to stimulate the tissue regeneration process. To understand the role that BG may have in muscle regeneration, in the following paragraphs, we will review the structure of BG, as well as the phenomenon that occurs when it is introduced into the body. BG was initially pioneered by Hench et al. in 1969 in the composition 45SiO_2_∙24.5Na_2_O∙24.5CaO∙6P_2_O_5_ (wt%), called 45S5 Bioglass^®^ [41]. In this case, the network former elements are silica (Si) and phosphorus (P), whereas the network modifiers are calcium (Ca) and sodium (Na). The silica glass structure is based on interconnected SiO_4_ tetrahedral units, and the phosphosilica glasses are based on PO_4_ and SiO_4_ tetrahedral units. By introducing the modifiers, depolymerization of the network occurs, and non-bridging oxygen appears [42].

**Figure 3:**
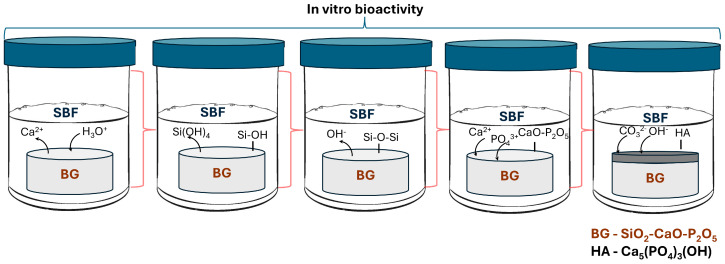
Schematic illustration of apatite layer formation on silicate glasses in body fluids. The process proceeds through five sequential stages. (1) Ion exchange: alkali and alkaline-earth ions (e.g., Ca^2+^) are released from the BG surface into SBF, while H^+^/H_3_O^+^ ions are incorporated, forming silanol (Si–OH) groups; (2) silica dissolution: hydrolysis of the glass network results in the release of soluble silica species (Si(OH)_4_) into the solution; (3) silica re-polymerization: silanol groups condense and re-polymerize to form a silica-rich layer composed of Si–O–Si bonds; (4) CaP deposition: calcium and phosphate ions from the SBF accumulate on the silica layer, forming an amorphous CaO–P_2_O_5_ phase; (5) HA formation: the amorphous phase crystallizes into hydroxyapatite, a mineral known to support cellular adhesion and tissue regeneration.

The original purpose of BG was to serve as a substitute for materials that were previously regarded as inert in the repair process of bone defects. A special property was that in the in vivo behavior, the BG can bond to bone via an apatite layer formed on the BG surface, promoting bone formation [43]. This phenomenon, called in vitro bioactivity, has been proven beneficial in soft tissue regeneration [44]. When bioactive glass comes into contact with body fluids, structural and chemical changes occur on the surface. In the in vitro bioactivity assay, the most accepted biological fluid is the simulated biological fluid (SBF) proposed by Kokubo [45,46]. Nevertheless, the assembled apatite layer on the BG surfaces appears after their immersion in phosphate buffer saline (PBS) [47], or Mueller–Hinton Broth (MHB) used in bacterial cultures [48]. The SBF is an acellular solution with pH and an ion concentration almost equal to human plasma. The apatite formation dynamics depend on the surface of the sample, the glass composite, the solution volume/glass surface area ratio, the calcium, and the phosphorus content [49,50,51]; however, all BG cover the same reaction steps [52,53,54,55] as follows (Figure 3):

- In the first stage, Na^+^, K^+^, or Ca^2+^ ions from glass are exchanges with H^+^, H_3_O^+^ from solution. The leached ions depend on the glass composition. Vallet-Regi et al. [56] demonstrated that in the SiO_2_-CaO binary glass systems, the calcium content in the solution increases during the first hours, which is a result of the hydrolysis of Si-O-Ca groups.
- In the second stage, silanization occurs; the soluble silica in the form of Si(OH)_4_ leaches into the solution by breaking Si-O-Si bonds, resulting in silanol (Si-OH).
- The third stage is the condensation and repolymerization of SiO_2_ by forming Si-O-Si bonds.
- In the fourth stage, an amorphous calcium phosphate layer forms through the migration of Ca^2+^ and PO_4_^3−^ ions to the silica-rich layer.
- In the last stage, the hydroxyapatite (HA) layer crystallization occurs by including the CO_3_^2−^ and OH^−^.

**Figure 4:**
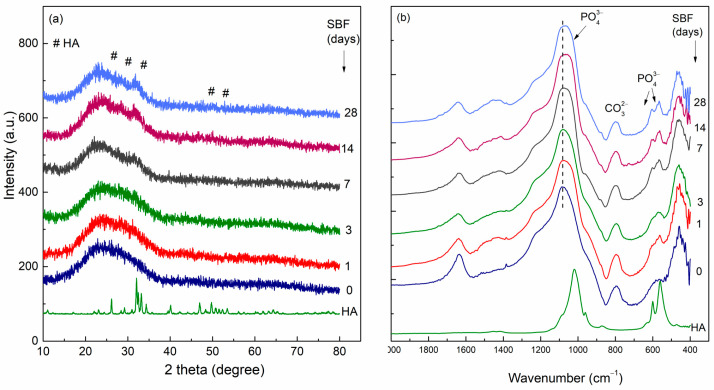
XRD pattern (**a**) and FT-IR spectra (**b**) of 70SiO_2_∙26CaO∙4P_2_O_5_ (mol%) bioactive glass before and after immersion in SBF for 1, 3, 7, 14, and 28 days at 37 °C.

Using X-ray diffractions (XRDs) and Fourier Transform Infrared (FT-IR) spectra measurements, we can follow the apatite formation on the BG surface over time after immersion in SBF (Figure 4). After 28 days of immersion (Figure 4a), the reflection of the HA crystalline phase is evidenced at 2θ = 25.9°, 28.3°, and 31.6° [57]. One can see the increase in these reflections over time. In the FT-IR spectra, we can see the doublet at 604 and 564 cm^−1^ assigned to the P-O bending vibration from the crystalline HA in all immersed samples [58]. Correlating this doublet with the Si-O-Si bending at 465 cm^−1^, the doublet shows an increase in time.

Analyzing the BG properties revealed the potential to be used in VML regeneration. Silicon ions play a crucial role in silicate-based BG, with Si^4+^ being the primary component released in body fluid, as mentioned above [59,60]. These ions play a significant role in stimulating collagen production, which is crucial for soft tissue regeneration [60,61]. Moreover, Si^4+^ has proven to be essential in skin repair by modulating collagen production, thereby potentially preventing scar tissue formation. Transforming growth factor-beta (TGF-b) is a growth factor known for increasing collagen production and supporting various stages of wound healing, including inflammation, proliferation, and tissue remodeling [62,63].

Calcium is a fundamental component of bones, capable of initiating bone remodeling [64]. It is essential for the proper functioning of nerves, cells, muscles, and bones. The Ca^2+^ cations are integral in cell activation mechanisms, regulating diverse growth-related processes and cell functionality [65]. Thus, releasing Ca^2+^ cations from BG can have a positive effect on cell mechanisms.

Biocompatibility and cytotoxicity are important factors in tissue regeneration applicability. Recent studies have explored BG’s potential in promoting myogenic differentiation and muscle tissue repair [66,67,68]. Winston et al. [67] investigated the biocompatibility of three BG nanoparticles, 60Si-BGN (60SiO_2_∙36CaO∙4P_2_O_5_ mol%), 80Si-BGN (80SiO_2_∙16CaO∙4P_2_O_5_ mol%), and 100Si-BGN (SiO_2_), by co-culturing them with C2C12 and L929 cell lines over 1, 3, and 5 days. Their results confirmed that both cell types well tolerated all three samples. Among them, 80Si-BGN stood out by significantly enhancing the proliferation of both cell lines after three days, underscoring its superior capacity to support cell growth. This effect was attributed to the optimal calcium content within the 80Si-BGN composition, which was identified as a key factor in stimulating cell proliferation. Boron-doped mesoporous bioactive glass nanoparticles (50SiO_2_∙40CaO∙10B_2_O_3_ and 45SiO_2_∙37CaO∙18B_2_O_3_ mol%) have demonstrated high biocompatibility with muscle cells. In vitro studies using C2C12 myoblasts showed that low concentrations (0.1 and 1 mg/mL) of these nanoparticles maintained high cell viability and promoted differentiation into myotubes. However, higher concentrations (10 mg/mL) resulted in reduced differentiation, indicating a dose-dependent response. The decrease in cell viability may be attributed to cytotoxic effects resulting from increased [BO_3_]^3−^ concentrations and elevated pH levels caused by boron substitution [66].

While BG are generally recognized for their biocompatibility, their cytotoxicity can vary depending on composition and concentration. High concentrations of BG nanoparticles may lead to increased oxidative stress and cytotoxicity. Therefore, optimizing the dosage and composition of BG is essential to minimize potential adverse effects while maximizing their regenerative capabilities.

Angiogenesis is a critical characteristic of materials used in tissue engineering. This property can be enhanced by incorporating elements such as Cu, Co, and Au, which are known for promoting blood vessel formation within the glass matrix [69,70,71,72]. BG stimulates angiogenesis through a multi-step process [73]. Upon implantation, it releases ions (additives or dopants) that trigger the production of vascular endothelial growth factor (VEGF), attracting endothelial cells. BG with a porous structure provides a scaffold for these newly migrating endothelial cells to attach and grow. Additionally, the surface chemistry of BG can be tailored to promote cell adhesion, further facilitating blood vessel formation. Newly formed blood vessels are vital for delivering oxygen and essential nutrients to regenerate tissue. Improved blood flow facilitated by angiogenesis promotes better implant integration with the surrounding tissue. This creates a more favorable environment for long-term tissue regeneration success [74,75,76,77,78].

VEGF, a potent angiogenic growth factor, is released by different kinds of cells [79,80]. Cardiomyocytes produce and release a variety of paracrine signaling molecules, including VEGF, basic fibroblast growth factor (bFGF), and platelet-derived growth factor (PDGF) [81,82]. A study revealed that low-dose BG (SiO_2_-CaO-P_2_O_5_) extracts enhanced VEGF gene expression in cardiomyocytes via the HIF-1α pathway [83].

However, when applied independently, BG encounters challenges, including difficulty sustaining ideal moisture levels and offering adequate biodegradability and bioadhesive strength for successful tissue regeneration. Thus, BG can be used as an active component in muscle regeneration by introducing it into polymer composites, resulting in an injectable suspension, paste, or hydrogel with different viscosities. Depending on the target tissue, either natural or synthetic polymers can be selected to fulfill the specific requirements for the desired tissue regeneration [84]. Frequently used polymers in this context encompass chitosan [85], collagen [86], gelatin [87], alginate [71], cellulose derivatives [88], poly (lactic-co-glycolic acid) (PLGA) [89], polyethylene glycol (PEG) [90], and polymethyl methacrylate (PMMA) [91]. The porosity, swelling ratio, and biodegradation of bioactive glass–polymer composites can be adjusted by combining two or more polymers. It is a challenge to achieve a degradation rate comparable to the regeneration of muscle tissue. Sergi et al. [92] summarized the advantages and disadvantages of different biopolymers such as collagen, gelatin, silk fibroin, hyaluronic acid, chitosan, alginate, and cellulose, and they summarized the influence of BG on the polymer’s mechanical structure. The synthesis methods are also a key factor regarding the composite’s mechanical properties [93]; for example, lyophilization can be used to obtain a composite with macropores [72], with three-dimensional (3D) printing technology that can mimic natural tissue properties [94]. Faster biodegradation was obtained by Zhang et al. [95] by synthesizing deferoxamine mesylate grafter alginate–BG (45S glass) hydrogel. The mass of this hydrogel began degrading on day 10, and only 48% of the original weight was maintained on day 14. Zhang et al. [96] studied the compressive modulus of the chitosan-sodium alginate/BG (58SiO_2_-33CaO-9P_2_O_5_, wt%) composites. They achieved that, after the addition of BG, the pore aperture of the composite increases, and its shape becomes irregular, resulting in a reduction of the compressive modulus, but it remains within the cartilage tissue application limit. Magyari et al. [97] obtained better wettability by introducing pullulan in the alginate–BG (60SiO_2_·32CaO·8P_2_O_5_ mol%) composite, obtaining a water uptake twice as high.

## 6. Bioactive Glass’s Role in Muscle Regeneration

The literature search focused mainly on articles that discuss BG in terms of muscle regeneration. A comprehensive search was performed on PubMed and Web of Science databases using combinations of the following keywords: “volumetric muscle loss”, “muscle regeneration”, “bioactive glasses”, “bioactive glass”, and “bioglass”. A search on PubMed and Scopus for articles with “bioactive glass” in the title, dating back to 1973, returned over 5700 results. Among these, more than 400 were review articles. The term “bioactive glass bone” yielded the highest number of articles, with over 3172 publications. In contrast, the keyword “bioactive glass muscle” returned just over 75 articles. Search terms including “volumetric muscle loss”, “muscle regeneration”, “bioactive glasses”, “bioactive glass”, and “bioglass” yielded few relevant publications, highlighting the limited available research in this area. The findings revealed limited information on the application of BG in muscle regeneration. Articles between the years 1973 and 2025 were considered. In the following paragraphs, we summarize the available data from these studies.

As already mentioned, BG was initially developed as a bone graft material, but it was later found to possess remarkable osteogenic, angiogenic, immunomodulatory, and antibacterial properties. These characteristics have expanded its potential applications, including its use in regenerating tissues and organs such as skin or muscle. Innovative use of biomaterials to transmit chemical and physical signals to muscle cells, or to serve as delivery systems for drugs and cells, presents exciting opportunities for muscle regeneration.

For skeletal muscle regeneration, Abou Neel et al. [98] proposed phosphate-based glass (P_2_O_5_-CaO-Na_2_O) fibers with Fe_2_O_3_ content, the role of Fe_2_O_3_ being myotube formation [99]. Analyzing the degradation rate and ion release, they concluded that phosphate-based glass fibers containing 3–5% Fe_2_O_3_ with a diameter of approximately 30 µm would be more durable as a scaffold for initial cell attachment. This is probably the first study to propose BG to skeletal muscle regeneration, but it is limited to in vitro assays. Following confirmation of myogenicity in the next study, the Fe_2_O_3_-containing phosphate-based glass disks were coated with collagen to enhance skeletal muscle cell behavior, obtaining comparable cellular metabolic activity to control [100]. It has been demonstrated that soluble phosphate-based glass fibers can support the proliferation and differentiation of human masseter muscle-derived cell cultures, being promising materials for muscle tissue regeneration. In another study, Shah et al. [101] demonstrated that phosphate-based glass (62.9P_2_O_5_∙21.9Al_2_O_3_∙15.2ZnO mol%) fibers can support the proliferation and differentiation of human masseter muscle-derived cell cultures.

**Figure 5:**
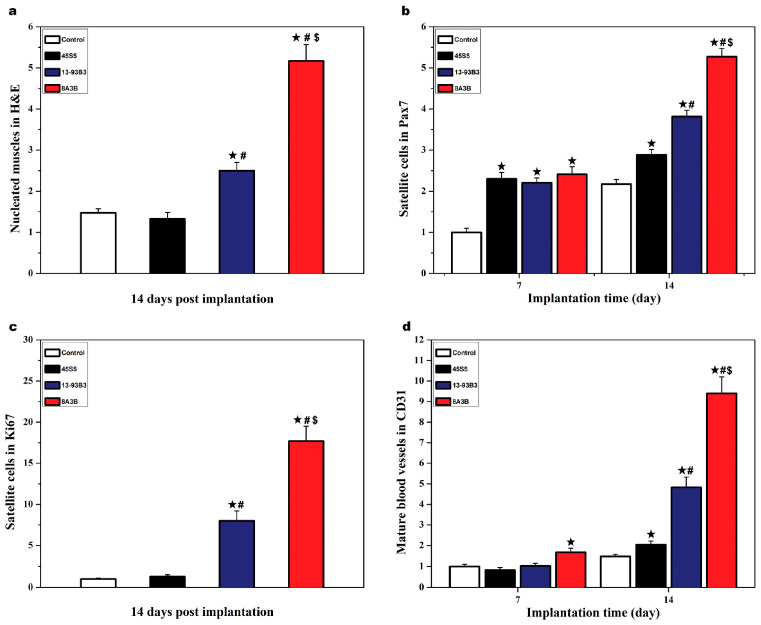
Quantitative comparison of control and bioactive glass groups at 7 and 14 days post operation: (**a**) nucleated muscles in H&E staining; (**b**) satellite cells in Pax 7 staining; (**c**) satellite cells in Ki67 staining; and (**d**) mature blood vessels in CD31 staining. (★ *p* < 0.05 compared to control group; # *p* < 0.05 compared to 45S5; $ *p* < 0.05 compared to 13-93B3). (Reprinted from Publication [68], page 314, Copyright (2019), with permission from Elsevier.).

Jia et al. [68] found that BG can activate the production of satellite cells and muscle stem cells without the incorporation of extra stem cells or growth factors. Satellite cells are skeletal muscle progenitor cells that are essential for skeletal muscle regeneration and contribute to the formation of new muscle fibers [102,103,104]. They investigated melt-derived 45S5 silicate (45SiO_2_∙24.5Na_2_O∙24.5CaO∙6P_2_O_5_ wt%), 13-93B3 borate (56.7B_2_O_3_∙5.5Na_2_O∙11.1K_2_O∙4.6MgO∙18.4CaO∙3.4P_2_O_5_ wt%), and 8A3B aluminoborate (50.7B_2_O_3_∙10.8Al_2_O_3_∙4.9Na_2_O∙9.9K_2_O∙4.1MgO∙16.4CaO∙3.2P_2_O_5_ wt%) glasses. In vitro tests indicated that the glasses could support angiogenesis and could simulate gene expression for muscle regeneration. Among the three types of glass, 8A3B aluminoborate showed better regenerative capabilities than 45S5 silicate or 13-93B3 borate glass, aligning with in vitro results (Figure 5). This initial 2-week study suggests the potential use of BG alone for skeletal muscle regeneration, likely due to the release of ions like B^3+^ and Ca^2+^ contributing to their biological performance.

**Figure 6:**
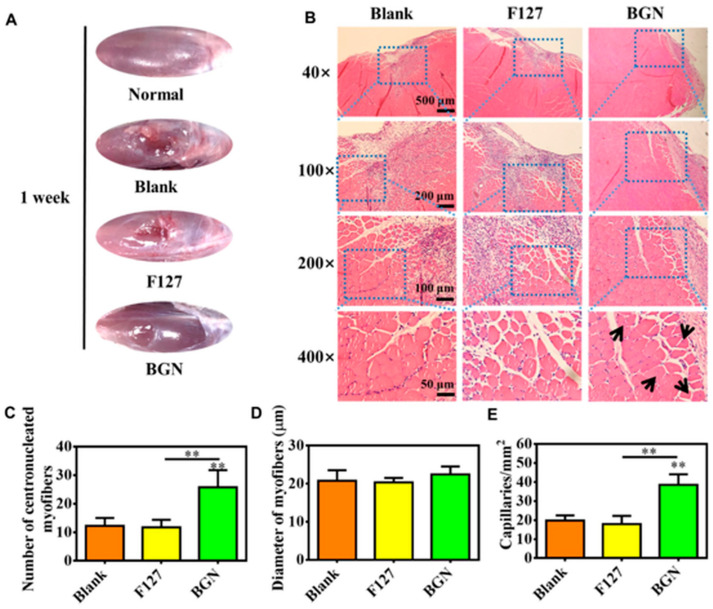
In vivo repair evaluation of tibialis anterior muscle defect in rats after 1 week. (**A**) Apparent observation of the skeletal tissue repair after 1 week; (**B**) H–E sections, staining images of muscle tissue after treating with different samples after 1 week; and calculated results on the number of centronucleated myofibers (**C**), diameter of myofibers (**D**), and density of capillaries (**E**). ** *p* < 0.01. Reprinted from Publication [67], page 7, Copyright (2023), with permission from Published by Oxford University Press).

For muscle tissue application, Ege et al. [66] propose the boron-doped mesoporous bioactive glass (SiO_2_-CaO-B_2_O_3_) nanoparticles. Mesoporous bioactive glass nanoparticles are attractive for several biomedical applications due to their ordered porosity and small size. It was found that the extract with these glasses induces differentiation of the myoblast (C2C12) cell line into myotubes, which is a promising property for muscle regeneration. The same promising results were obtained by Windston et al. [67] on silicate (SiO_2_-CaO-P_2_O_5_) glass nanoparticles (BGNs), namely these glass nanoparticles can stimulate the myogenic differentiation of C1C12 cells, leading to both myotube formation and increased expression of myogenic genes. For applicability, BGNs were introduced in Pluronic F-127 to obtain thermal-responsive nanocomposite hydrogels. The in vivo results showed that the rat skeletal muscle defect with the used composite regenerated fully within a 4-week implantation period (Figure 6). This study highlights the critical influence of BGN composition, particularly the silicon-to-calcium ratio, on skeletal muscle repair.

Recently, Lu et al. [105] demonstrated the promising role of Zn-containing BG (SiO_2_–CaO-ZnO) in enhancing the 3D printing properties of alginate-dialdehyde-gelatin-based hydrogels and cell proliferation. The role of BG in biopolymers or hydrogels is bioactive inorganic fillers, acting as carriers for therapeutic ions. For this reason, the studied hydrogels are considered for future investigations of ion-loaded bio-inks for muscle tissue engineering.

Table 1 summarizes the various properties of BGs and their applicability in muscle tissue regeneration.

## 7. Conclusions and Future Perspectives

Ongoing clinical trials related to BG are likely to pave the way for the approval of more products in the near future. Since many composites with BG for soft tissue regeneration are still in the early phases of development, further investigation into their mechanisms of action, particularly for neural and muscle regeneration, will be essential. The therapeutic efficacy of BG-based strategies for soft tissue regeneration is largely attributed to the dissolution products of BG. Therefore, future research should concentrate on elucidating how the individual and synergistic effects of the ions released from BG influence critical physiological processes, such as angiogenesis and immune responses.

Muscle regeneration in adult tissue is a vital homeostatic process requiring precise regulation to ensure functional recovery and prevent pathological alterations. This involves the coordinated activation of multiple biological factors.

Overall, the use of BG with polymers for muscle regeneration holds significant promise in future testing trials for improving functional recovery after substantial muscle loss. This review article provides an overview of VML, the mechanisms of muscle regeneration, and the emerging role of biomaterials in promoting successful tissue repair. The integration of BG into the realm of muscle regeneration and VML treatment presents a convincing option that leads to significant changes. In the context of muscle regeneration, BG can be used as a composite component, or as coatings that release bioactive ions, such as calcium and phosphate, into the surrounding tissue environment. These ions can stimulate cellular responses, including angiogenic (blood vessel formation) activities. While BG are more commonly associated with bone tissue engineering, due to its osteo-inductive properties, it has also been explored for its potential to enhance muscle regeneration. Matching the mechanical and structural features of targeted soft tissues is essential for BG composites to support effective healing without complications. However, current research highlights a persistent gap between the mechanical properties of developed BG composites and the requirements for soft tissue regeneration. Therefore, further experiments are necessary to achieve optimal composition, structure, strength, and biodegradability. Additionally, extensive in vitro and in vivo testing is required before advancing to clinical trials or potential commercialization. The continued interdisciplinary collaboration between researchers, clinicians, and engineers is essential to refine and translate these biomaterial-based strategies into effective clinical treatments for muscle regeneration after VML.

**Figure 7:**
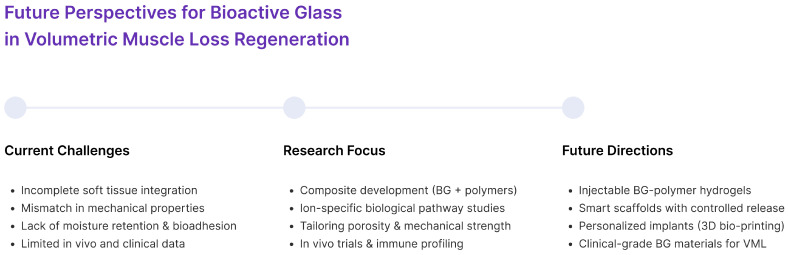
Schematic illustration of the future perspective of BG in VML.

A major objective in BG research is the design of materials capable of serving as hard tissue replacements while simultaneously enhancing tissue regeneration. In Figure 7, several possible research directions for obtaining materials applicable in VML regeneration have been suggested.

In conclusion, BG has expanded its role beyond a bone graft material to become a versatile and promising option for regenerating a range of soft and hard tissues and organs. The unique properties of BG, including its ability to promote osseointegration, angiogenesis, antibacterial effects, and immunomodulation, have made it an attractive clinical material, supported by decades of clinical data confirming its safety since around 1970.

## Figures and Tables

**Table 1:** Different BG compositions considered for skeletal muscle tissue engineering.

Bioactive Glasses	Cell Type	Applied Form	Advantages/Disadvantages	Ref
50P_2_O_5_∙30CaO∙(20-x)Na_2_O∙xFe_2_O_3_ (mol%) x = 0, 1, 2, 3, 4, 5	Human craniofacial (masseter) muscle cell cultures	collagen coated glass fibers (in vitro)	Low toxicity to human craniofacial skeletal muscle cells, supporting cell proliferation and differentiation. The use of aligned collagen-coated Fe5-mol% glass fibers provided essential biophysical cues, promoting unidirectional cell alignment and enhancing the expression of adult MYH genes. The degradation products of phosphate-based glasses can be acidic, potentially leading to local inflammation and affecting tissue regeneration.	[98,100]
62.9P_2_O_5_ 21.9Al_2_O_3_ 15.2ZnO	Human masseter muscle-derived cell cultures	Glass fibres (in vitro)	Soluble phosphate-based glass fibers supported the proliferation and differentiation of human masseter muscle-derived cell cultures.	[101]
45S5 (45SiO_2_∙24.5Na_2_O∙24.5CaO∙6P_2_O_5_ wt%), 13-93B3 (56.7B_2_O_3_∙5.5Na_2_O∙11.1K_2_O∙4.6MgO∙18.4CaO∙3.4P_2_O_5_ wt%), 8A3B (50.7B_2_O_3_∙10.8Al_2_O_3_∙4.9Na_2_O∙9.9K_2_O∙4.1MgO∙16.4CaO∙3.2P_2_O_5_ wt%)	Mouse myoblast C2C12	BG particles (in vivo)	Stimulated angiogenesis and boosted secretion of muscle-relevant growth factors (e.g., IGF-1). 8A3B glass supported muscle repair in vivo without the need for added growth factors or stem cells.	[68]
MBG (58SiO_2_-42CaO mol%), 10B-MBG (50SiO_2_-40CaO-10B_2_O_3_ mol%), 18B-MBG (45SiO_2_-37CaO-18B_2_O_3_ mol%)	Myoblast C2C12	Mesoporous BG (in vitro)	Enhanced myogenic differentiation at low concentrations (0.1–1 mg/mL). High cell viability; mesoporous structure with negative surface charge favorable for cell interaction. Reduced differentiation at higher concentration (10 mg/mL).	[66]
100Si-BGN (SiO_2_), 80Si-BGN (80SiO_2_-16CaO-4P_2_O_5_ mol%), 60Si-BGN (60SiO_2_-30CaO-4P_2_O_5_ mol%),	Myoblast C2C12, Fibroblast L929	BG-Pluronic F127 hydrogel (in vivo)	80Si-BGN significantly promoted myogenic differentiation of C2C12 cells, evidenced by increased myotube formation and elevated expression of myogenic markers. 80Si-BGN enhanced skeletal muscle regeneration in a rat tibialis anterior muscle defect model, leading to increased centronucleated myofiber formation and higher capillary density after 4 weeks of implantation.	[67]
8020 (80SiO_2_–20CaO mol%), 2Zn (80SiO_2_–18CaO-2ZnO mol%), 5Zn (80SiO_2_–15CaO-5ZnO mol%), 10Zn (80SiO_2_–10CaO-10ZnO mol%)	Myoblast cell line C2C12 cells	BG-alginate-gelatin hydrogels (in vitro)	Controlled; Zn modulated Si/Ca release. Promotes C2C12 cell proliferation, spreading, elongation, and alignment. Zinc ions in the BG nanoparticles helped regulate silicon and calcium release, avoiding a rapid surge and creating a stable environment for cell growth and differentiation.	[105]

## Data Availability

No new data were created or analyzed in this study. Data sharing is not applicable to this article.

## Abbreviations

The following abbreviations are used in this manuscript:

VML	Volumetric Muscle Loss
BG	Bioactive glasses
VGEF	Vascular endothelial growth factor
bFGF	Linear dichroism basic fibroblast growth factor
PDGF	platelet-derived growth factor
